# Analysis of the Catecholaminergic Phenotype in Human SH-SY5Y and BE(2)-M17 Neuroblastoma Cell Lines upon Differentiation

**DOI:** 10.1371/journal.pone.0136769

**Published:** 2015-08-28

**Authors:** Roberta Filograna, Laura Civiero, Vanni Ferrari, Gaia Codolo, Elisa Greggio, Luigi Bubacco, Mariano Beltramini, Marco Bisaglia

**Affiliations:** 1 Molecular Physiology and Biophysics Unit, Department of Biology, University of Padova, Padova, Italy; 2 General Pathology Unit, Department of Biology, University of Padova, Padova, Italy; University of Navarra, SPAIN

## Abstract

Human cell lines are often used to investigate cellular pathways relevant for physiological or pathological processes or to evaluate cell toxicity or protection induced by different compounds, including potential drugs. In this study, we analyzed and compared the differentiating activities of three agents (retinoic acid, staurosporine and 12-O-tetradecanoylphorbol-13-acetate) on the human neuroblastoma SH-SY5Y and BE(2)-M17 cell lines; the first cell line is largely used in the field of neuroscience, while the second is still poorly characterized. After evaluating their effects in terms of cell proliferation and morphology, we investigated their catecholaminergic properties by assessing the expression profiles of the major genes involved in catecholamine synthesis and storage and the cellular concentrations of the neurotransmitters dopamine and noradrenaline. Our results demonstrate that the two cell lines possess similar abilities to differentiate and acquire a neuron-like morphology. The most evident effects in SH-SY5Y cells were observed in the presence of staurosporine, while in BE(2)-M17 cells, retinoic acid induced the strongest effects. Undifferentiated SH-SY5Y and BE(2)-M17 cells are characterized by the production of both NA and DA, but their levels are considerably higher in BE(2)-M17 cells. Moreover, the NAergic phenotype appears to be more pronounced in SH-SY5Y cells, while BE(2)-M17 cells have a more prominent DAergic phenotype. Finally, the catecholamine concentration strongly increases upon differentiation induced by staurosporine in both cell lines. In conclusion, in this work the catecholaminergic phenotype of the human BE(2)-M17 cell line upon differentiation was characterized for the first time. Our data suggest that SH-SY5Y and BE(2)-M17 represent two alternative cell models for the neuroscience field.

## Introduction

In the vertebrate central nervous system, catecholaminergic (CAergic) neurons constitute anatomically discrete groups of cells that synthesize and release the neurotransmitters dopamine (DA) and noradrenaline (NA). DAergic neurons, which originate in the ventral tegmental area, the substantia nigra and the hypothalamus, are involved in motor control, the control of emotional balance, reward-associated behavior, attention, and memory and the secretion of hormones such as prolactin [1] The majority of NA neurons are concentrated in the locus coeruleus and contribute to the regulation of arousal, sleep–wake patterns, sensory perception and emotional status [2, 3]. Considering the numerous functions attributed to the activity of CAergic neurons, it is not surprising that this class of cells is associated with multiple neurodegenerative, psychiatric, and endocrine disorders. For example, the selective degeneration of DAergic neurons in the substantia nigra is associated with the trembling and muscular rigidity that are symptomatic of Parkinson’s disease. A malfunction in the mesocortical and mesolimbic DAergic pathways is linked to schizophrenia and the attention deficit, addiction, and hyperactivity disorders. Dysregulation of the NAergic system may result in deficits in a variety of cognitive and affective processes and is related to depression and sleep disorders.

A number of cellular models have been described to gain insight into the molecular pathways that are dysfunctional in CAergic-related disorders and to investigate potential therapeutic agents. Among them, human neuroblastoma cell lines have been used as *in vitro* models for the study of the mechanisms of action and neurotoxicity of compounds on the nervous system [4]. Furthermore, neuroblastoma cell lines can be differentiated with chemicals or growth factors supplied to the cultured medium. Differentiation arrests cell division and induces morphological changes that are characteristic of neurons, including the extension of neurites. Importantly, these cells have been reported to release neurotransmitters under depolarizing conditions [5–9].

The human neuroblastoma SH-SY5Y cell line (ATCC CRL-2266) has been largely used in the field of neuroscience, particularly to generate different Parkinson’s disease cell models [10–12]. These cells, which were subcloned from the SK-N-SH cell line, are of neuronal origin, express tyrosine hydroxylase (TH) and exhibit moderate levels of dopamine-β-hydroxylase (DßH) activity, which is specific for NAergic neurons [13]. A variety of agents, including retinoic acid (RA) [12, 14], phorbol ester 12-O-tetradecanoylphorbol-13-acetate (TPA) [15, 16], brain-derived neurotrophic factor [17], dibutyryl cyclic AMP [18] and staurosporine [19], have been used to induce differentiation. In contrast to SH-SY5Y cells, much less is known about the BE(2)-M17 cell line (ATCC CRL-2267). These cells were cloned from the SK-N-BE(2) neuroblastoma cell line isolated from a 2-year-old male. Although the first biochemical characterization of these cells dates back to the 1980s [20, 21], their use has been limited. Differentiation with RA has been shown to induce morphological and metabolic changes that confer neuronal-like features [7, 22]. However, little is known about the CAergic pathway of BE(2)-M17 cells in the undifferentiated and differentiated states.

In this study, we compared the differentiating activities of three agents (RA, staurosporine and TPA) on SH-SY5Y and BE(2)-M17 cells; these agents were selected on the basis of their previous characterization in the SH-SY5Y cell line and ease of manipulation. Specifically, we analyzed a number of morphological properties, including the expression of the neuron-specific proteins β-tubulin III and neurofilament. Then, we focused on the CAergic pathway by evaluating the expression profiles of the major genes involved in CA synthesis and storage and the presence of DA and NA upon differentiation. Our results emphasize that the two cell lines tested possess similar abilities to differentiate and acquire neuron-like morphology. The most evident effects in the SH-SY5Y cells were observed in the presence of staurosporine, while RA induced the strongest effects in the BE(2)-M17 cells. There were some relevant differences in the CAergic pathway between the two cell lines. Undifferentiated SH-SY5Y cells produce both NA and DA, but the NAergic phenotype appears to be more pronounced. The CA concentration is strongly increased after staurosporine-induced differentiation and the cells become mostly NAergic. Undifferentiated BE(2)-M17 cells produce both NA and DA, but their amounts are significantly higher compared with those produced by SH-SY5Y cells and their phenotype is more DAergic. The CA concentration in these cells is also strongly increased upon differentiation with staurosporine.

## Materials and Methods

### Reagents

Tissue culture reagents were purchased from Gibco/Life Technologies. Chemicals were obtained from Sigma-Aldrich. Stock solutions of RA were prepared by dissolving the powder in DMSO, and TPA and staurosporine were dissolved in 100% ethanol. In all of the experiments, the final concentration of ethanol never exceeded 0.1% and had no detectable effect on cell growth or differentiation. The SH-SY5Y and BE(2)-M17 cell lines were purchased from the Cell Factory-IST Genova and LGC standards, respectively.

### Cell culture and differentiation

Undifferentiated human neuroblastoma SH-SY5Y and BE(2)-M17 cells were maintained in a 1:1 mixture of Ham's F12 and Dulbecco’s Modified Eagle’s Medium supplemented with 10% fetal bovine serum and grown in the presence of 5% CO_2_ in a humidified incubator at 37°C. The cell medium was replaced every 3 days, and the cells were sub-cultured once confluence was reached. In all of the experiments, the cells were used at early passages (P1-5 after purchase). Differences in morphology between proliferative and differentiated cells were evaluated by phase contrast light microscopy (Motic AE2000)

For cell proliferation analysis, 1×10^5^ cells were seeded into 25 cm^2^ flasks. Twenty-four hours after seeding, differentiation was induced by the addition of TPA, RA or staurosporine at concentrations of 15 nM, 10 μM or 10 nM for SH-SY5Y cells and 30 nM, 5 μM or 8 nM for BE(2)-M17 cells, respectively. Fresh media containing the specified inducing agent was provided every 2 days. To determine the rate of cell growth, cells were harvested after a 0.05% trypsin treatment and quantified using a hemocytometer. Briefly, approximately 5–10 μl of the cell suspension was added into the Bürker chamber. Cells were counted in four large 1 mm^2^ squares under an inverted phase contrast microscope using a 10X magnification. Then, the average number of cells per square was determined. The total number of cells overlying a 1 mm^2^ square was in the range of 30–150.

### Immunocytochemistry and immunofluorescence

Cells were plated at a density of 2×10^4^ cells/coverslip in 2 ml of medium on 15 mm glass coverslips pre-coated with poly-D-lysine. After 7 days of differentiation, the cells were fixed for 30 min at room temperature with 4% paraformaldehyde in a phosphate buffered saline (PBS) solution (pH 7.4), rinsed three times with PBS, permeabilized with 0.1% Triton X-100 in PBS for 30 min at room temperature and incubated in blocking solution (5% fetal bovine serum in PBS) for 30 min at room temperature. The cells were incubated for 1 hour at room temperature with primary antibodies diluted in blocking solution. The primary antibodies used in this study included mouse anti-neurofilament (1:1000, Covance) and mouse anti-neuron specific β-III tubulin (1:200, Sigma). After 3 washes in PBS, the cells were subsequently incubated with secondary anti-mouse antibodies conjugated with Alexa Fluor-488 and -568 (Life technologies) at a 1:200 dilution for 1 hour at room temperature. Nuclei were counterstained using 0.16 μM Hoechst 33258 (Gibco/Life Technologies) for 5 min, and after extensive washing in PBS the coverslips were mounted with ProLong Gold Antifade (Life Technologies). Images were acquired using a Leica DM5000 epifluorescence microscope.

### Neuritic outgrowth

To measure neuritic outgrowth after differentiation, SH-SY5Y and BE(2)-M17 cells were seeded onto coverslips pre-coated with poly-D-lysine. After 24 hours, the cells were transfected with a vector containing the coding sequence for a cytosolic green fluorescent protein whose expression allowed the neurite length to be tracked at the single cell level using a fluorescence microscope. The day after transfection, differentiation was induced as described above. After 7 days of treatment, the samples were fixed; approximately 30 cells were analyzed for each condition. Images were acquired using a Leica 5000B epifluorescence microscope with a magnification of 20X. Neurite length was assessed using ImageJ software.

### mRNA expression analysis using semi-quantitative and quantitative real-time PCR

The determination of gene expression levels was performed using quantitative real-time PCR (qRT-PCR). Total RNA was extracted from undifferentiated or differentiated cells using TRIzol according to the manufacturer’s instructions (Gibco/Life Technologies). Reverse transcription was performed using the ImProm II Reverse Transcription System (Promega), and cDNA was obtained for the qRT-PCR reactions. Semi-quantitative PCR was performed with the GoTaq DNA Polymerase (Promega) using the following conditions for amplification: a single denaturation step at 95°C for 5 min, followed by 30 cycles of 30 s of denaturation at 95°C, 30 s of annealing at 60°C and 1 min of extension at 72°C, and a final extension step at 72°C for 10 min. Amplified DNA was subsequently analyzed through 2% agarose gel electrophoresis, and the images were acquired using the Gel Doc XR System (Bio-Rad). For quantitative analysis, qRT-PCR assays were performed in 96-well optical plates with a 7500 real-time PCR system (Applied Biosystems) using the following parameters: 95°C for 10 min, 38 cycles of 20 s at 95°C and 60 s at 60°C, followed by 2 cycles of 15 s at 95°C and 60 s at 60°C. cDNA was amplified using the Power SYBR Green Master Mix (Applied Biosystems) containing 0.2 μM of the primers. The primer sequences (forward and reverse) and the expected lengths of the amplified products are listed in Table 1. The expression of individual target genes was calculated using the ΔΔC_t_-method [23]. Sample C_t_−values were normalized by C_t_−values of the housekeeping genes. In the SH-SY5Y cells, glyceraldehyde 3-phosphate dehydrogenase (*GAPDH*) was the reference gene used for aromatic L-amino acid decarboxylase (*AADC*) and dopamine-β-hydroxylase (*DβH*) quantification, while RNA polymerase II (*RPII*) was used for tyrosine hydroxylase *(TH*) and vesicular monoamine transporter 2 (*VMAT2*). In the BE(2)-M17 cells, *GAPDH* was the internal control gene used for *AADC* quantification, while RNA polymerase II was used for *TH*, *VMAT2* and *DβH*. These two housekeeping genes were expressed at the same levels as the target genes. These results were further normalized using undifferentiated cells. For each condition tested, three technical replicates were performed and the average value was determined.

**Table 1:** List of primers used for qRT-PCR.

Gene	Primer sequence (5'—3')	Product length (bp)
*GAPDH*	Fw: ATGAAGGGGTCATTGATGG	138
	Rv: AAGGTGAAGGTCGGAGTCAA	
*RPII*	Fw-TTGGTGACGACTTGAACTGC	123
	Rv-CCATCTTGTCCACCACCTCT	
*TH*	Fw: GCCCTACCAAGACCAGACGTA	89
	Rv: CGTGAGGCATAGCTCCTGA	
*AADC*	Fw: GAAGCCCTGGAGAGAGACAA	121
	Rv: CCTTGTTGCAGATAGGACCG	
*VMAT2*	Fw: TGAAGAGAGAGGCAACGTCA	149
	Rv: CGTCTTCCCCACAAACTCAT	
*DβH*	Fw: GCCTTCATCCTCACTGGCTA	109
	Rv: TTCTCCCAGTCAGGTGTGTG	

### Quantification of catecholamine levels

Undifferentiated and TPA-, RA- or staurosporine-treated cells were harvested, washed with PBS, and mixed with ice-chilled 0.2 M perchloric acid containing 5 mM EDTA and 5 mM sodium bisulfate (250 μl of cell lysis solution was used for every 10^7^ cells). Lysates were centrifuged at 7000 x *g* for 20 min at 4°C, and the supernatants were collected and stored immediately at -80°C prior to analysis. High-performance liquid chromatography (HPLC) (Agilent 1100 Series) coupled with an ESA Coulochem II electrochemical detector was used to measure the NA and DA concentrations. Separations were achieved on a 150 × 4.6 mm Waters C18 column. The mobile phase consisted of 75 mM NaH_2_PO_4_, 1.7 mM 1-octanesulfonic acid, 25 μM EDTA and 10% (v/v) acetonitrile adjusted to pH 3 with phosphoric acid. The column was maintained at room temperature, and the flow rate was 0.6 ml/min. An analytical cell ESA 5011A was used with the electrochemical potentials set at -150 mV and +220 mV. The working standard solution was prepared in 0.2 M perchloric acid containing 5 mM EDTA and 5 mM sodium bisulfite. Five- to twenty-microliter samples were injected. The peak areas of the external standards were used to quantify the sample peaks. At least two different dilutions were injected for each condition analyzed, and each sample dilution was run in duplicate. The average value was determined after normalizing the concentration values with the appropriate dilution factors. For each sample used, the protein concentrations were detected using the BCA protein assay kit (Thermo Scientific Pierce); the DA and NA concentrations were expressed in nanomoles per gram of proteins.

### Statistical Analysis

Each experiment was performed in triplicate. The data were expressed as the mean ± S.E.M. Error propagation was used when required. Student’s t-test was used to evaluate statistically significant differences using the GraphPad Prism software.

## Results

### Effect of differentiation on growth inhibition

To identify the optimal experimental conditions for differentiation, we performed a preliminary analysis with both SH-SY5Y and BE(2)-M17 cells in which growth inhibition induced by various concentrations of each agent was tested. Starting from the values reported in previous studies on SH-SY5Y cells [14, 16, 19], the following ranges of concentration were tested: 1.5–150 nM for TPA, 1–50 μM for RA and 3–15 nM for staurosporine. Consistent with previous reports for SH-SY5Y cells, we observed the most pronounced effects on growth inhibition with 15 nM TPA, 10 μM RA and 10 nM staurosporine. For BE(2)-M17 cells, the most effective concentrations were 30 nM TPA, 5 μM RA and 8 nM staurosporine, which were close to the values reported above for SH-SY5Y cells.

**Fig 1:**
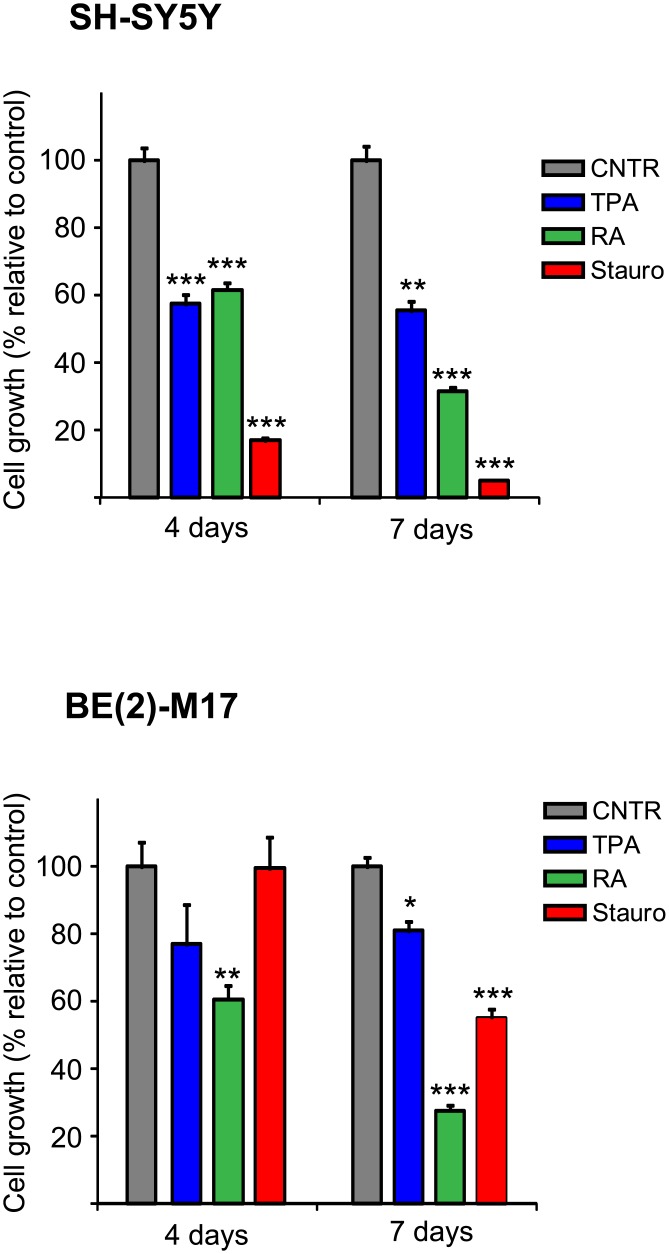
Cellular growth of undifferentiated and differentiated SH-SY5Y and BE(2)-M17 cells. All of the examined drugs inhibited cell proliferation. In SH-SY5Y cells the effect of staurosporine was the most pronounced, while in BE(2)-M17 cells the strongest inhibition was achieved with RA treatment. The number of cells is shown as the mean ± S.E.M. of three experiments. Differences between differentiated and undifferentiated cells were tested for significance using Student’s t-test. (*P<0.*05*, **P<0.*01*, ***P<0.*001)*.

Then, the cells were treated with the optimized concentration of each differentiating agent. Cell inhibition was subsequently assessed after 4 and 7 days of treatment after verifying that none of the differentiating agents induced apoptosis or necrosis in our cell models at the experimental concentrations used, as determined by cytofluorimetric analysis (S1 File). As shown in Fig 1, all agents induced growth inhibition, albeit with different efficiencies that were dependent on the type of treatment and the cell line used. In the case of SH-SY5Y, after 4 days the inhibition levels in the presence of RA and TPA were very similar (~40%), whereas RA promoted a more marked effect compared to TPA after 7 days (~70% and ~45%, respectively). Interestingly, staurosporine induced the most pronounced inhibition. After 4 days of treatment, cell proliferation was almost completely blocked, with a growth inhibition of ~80%; this inhibition was even more evident after 7 days (~95%).

The results obtained with BE(2)-M17 cells were quite different. There was almost no effect after 4 days of staurosporine treatment, while the growth inhibition in the presence of TPA reached ~20%. The strongest inhibition (~40%) was obtained in the presence of RA. After 7 days, staurosporine promoted a more marked effect compared to TPA (~45% and ~20%, respectively), although it was still low compared to the effects observed in the SH-SY5Y cells. RA induced the strongest inhibition (~70%) after 7 days. In conclusion, staurosporine was by far the most efficient treatment to inhibit cell growth in the SH-SY5Y cell line, while treatment with RA induced the most effective results in the BE(2)-M17 cell line.

### Morphological differentiation

**Fig 2:**
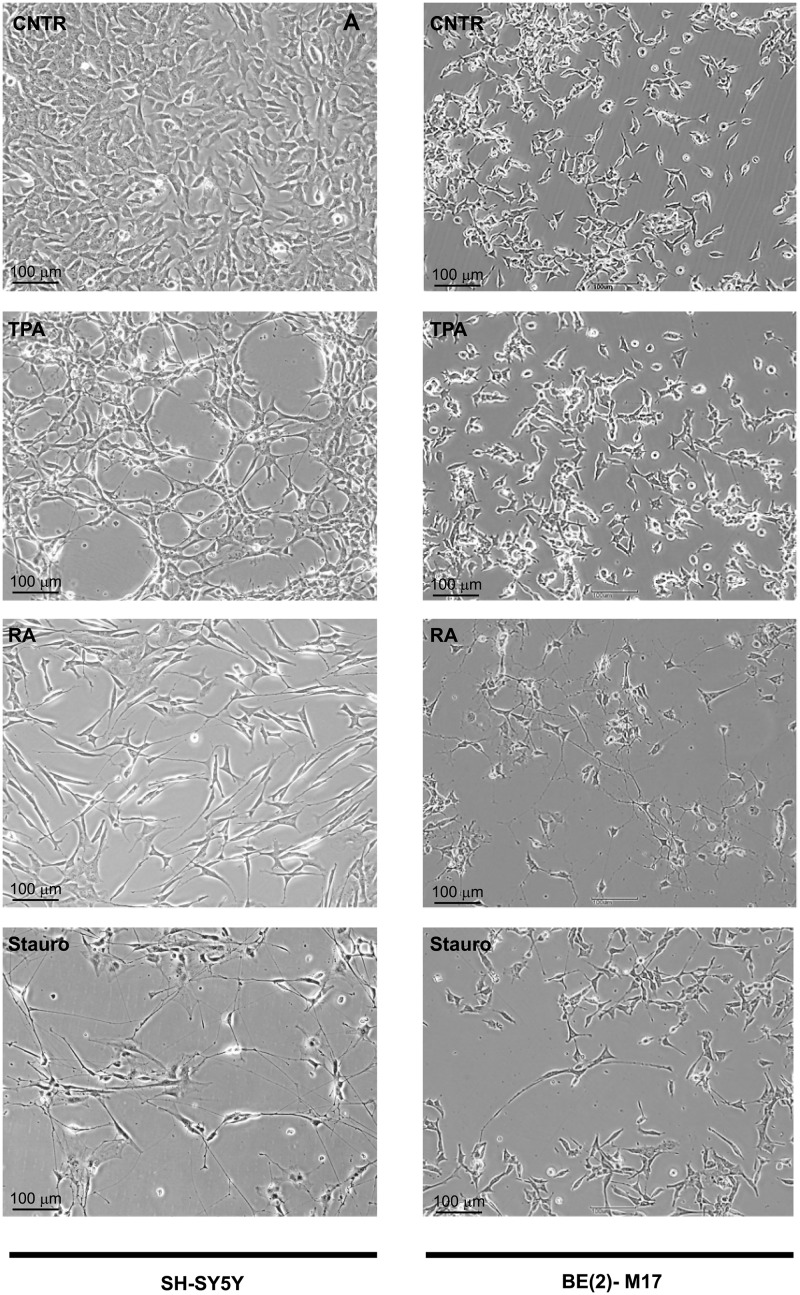
Cellular morphology of undifferentiated and differentiated SH-SY5Y and BE(2)-M17 cells after 7 days of differentiation. Representative phase contrast images showed that after 7 days of treatment, staurosporine and RA promoted the most remarkable neurite extensions in SH-SY5Y cells and BE(2)-M17 cells, respectively. Scale bar = 100 μM.

Next, we explored the effect of the three differentiating agents on cell morphology. Undifferentiated SH-SY5Y neuroblastoma cells displayed a characteristic morphology with rounded cell bodies and few short processes (Fig 2). A similar morphology was also present in the BE(2)-M17 cells, although they were smaller and the processes were less evident. After 7 days of treatment the cells displayed different levels of neurite lengths and numbers depending on the chemical used. Differentiation induced by TPA in both cell lines resulted in the formation of fewer short processes. Instead, both RA- and staurosporine-differentiated cultures showed a clear neuronal-like morphology with a complex network of neuritic extensions. In the SH-SY5Y cells, these processes were longer and more numerous after treatment with staurosporine compared to treatment with other differentiating agents. Conversely, in the BE(2)-M17 cells a more pronounced morphological differentiation was observed upon addition of RA.

**Fig 3:**
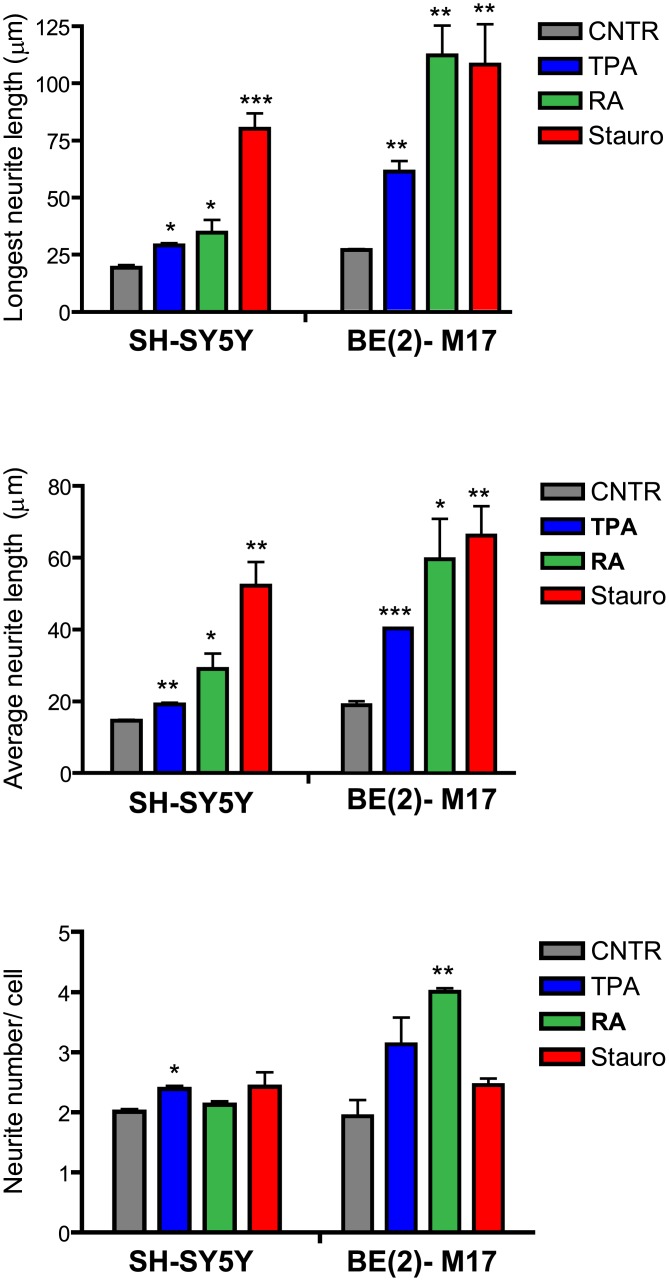
Neurite outgrowth in undifferentiated and differentiated SH-SY5Y and BE(2)-M17 cells after 7 days of differentiation. Staurosporine and RA promoted the most remarkable increase in neurite length in the SH-SY5Y cells and BE(2)-M17 cells, respectively. RA treatment also increased neurite branching in BE(2)-M17 cells. Data are expressed as the mean ± SEM of three experiments. At least 90 cells were analyzed for each conditions. Differences between differentiated and undifferentiated cells were tested for significance using Student’s t-test. (*P<0.*05*, **P<0.*01*, ***P<0.*001)*.

To better characterize the morphological changes and to quantify the effect of each differentiating agent in terms of neurite outgrowth, cells were transfected with a cytosolic green fluorescent protein that allowed neurites to be tracked at a single-cell level. Three parameters were used for the analysis: i) the length of the longest neurite; ii) the average length of the neurites; and iii) the number of neurites per cell. The results are summarized in Fig 3. In the SH-SY5Y cells, TPA and RA slightly increased both the average length of the neurites and the maximum neurite length compared with undifferentiated cells. Staurosporine was the more effective agent in terms of both the average and maximum length. None of the differentiating agents strongly impacted the number of neurites per cell, although there was a slight increase in the presence of TPA. BE(2)-M17 cells showed stimulated neuritic outgrowth for each agent tested after 7 days of treatment. The best morphological differentiation was accomplished by the addition of RA, which not only increased neurite length but also neurite number.

### Immunofluorescence analysis of neuronal markers

**Fig 4:**
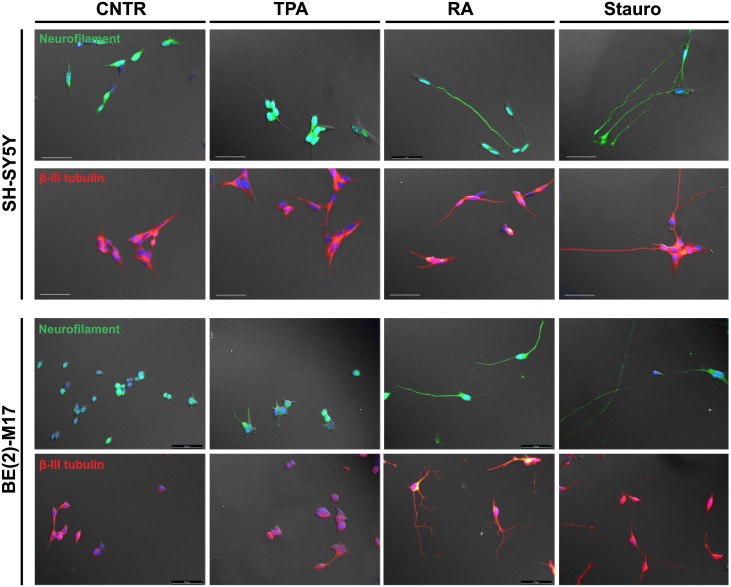
Immunofluorescence staining for the neuronal markers neurofilament and β-III tubulin. The comparison was made between undifferentiated and differentiated SH-SY5Y and BE(2)-M17 cells after 7 days of treatment. RA and staurosporine differentiation induced the formation of long processes positive for neurofilament and β-III tubulin in both cell lines. Blue: Hoechst; Green: neurofilaments; Red: β-III tubulin; Gray: phase contrast. Scale bar = 50 μM.

To evaluate whether differentiated cells expressed neuronal markers, we stained undifferentiated and differentiated SH-SY5Y and BE(2)-M17 cells with antibodies against neuron-specific proteins such as β-tubulin III and neurofilament. Class III β-tubulin is a microtubule element that is expressed exclusively in neurons, while neurofilaments are types of intermediate filaments present in axons. Consistent with our previous observations, RA and staurosporine but not TPA promoted marked variations in cell morphology in both the SH-the SY5Y and BE(2)-M17 cell lines, inducing a neuron-like appearance (Fig 4). TPA-treated cells showed only moderate neurite outgrowth and did not exhibit detectable neuronal-marker-positive processes. In contrast, RA- and staurosporine-differentiated SH-SY5Y and BE(2)-M17 cells showed a significant increase in β-tubulin III and neurofilament expression compared to undifferentiated cells.

### Gene expression profile

**Fig 5:**
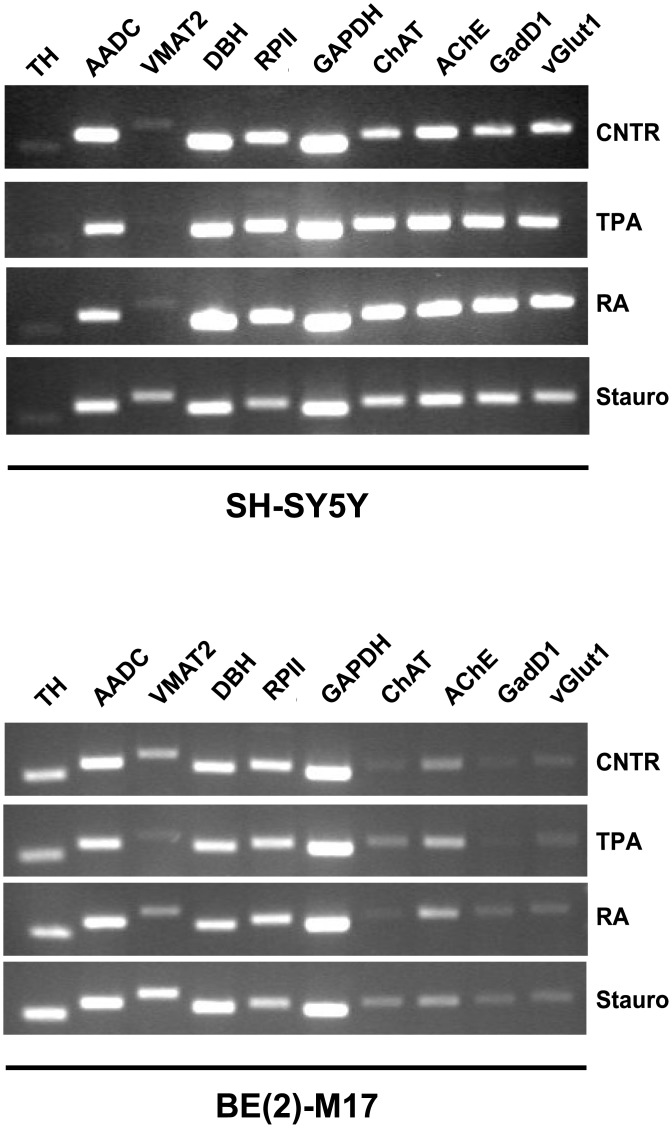
Gene expression profile of DAergic, cholinergic and glutamatergic markers in SH-SY5Y and BE(2)-M17 cells. TH, AADC, VMAT2, and DβH mRNAs were analyzed in undifferentiated cells and after 7 days of treatment with TPA, RA and staurosporine using semi-quantitative RT-PCR.

To analyze the effects of differentiation on the CAergic pathway in both SH-SY5Y and BE(2)-M17 cells, we evaluated variations in the gene expression profile before and after differentiation. Specifically, we focused on the key genes involved in CA synthesis and storage: TH and AADC are both involved in the synthesis of DA; VMAT2 rapidly sequesters DA from the cytosol into synaptic vesicles; and DβH synthesizes NA from DA inside synaptic vesicles. Because immature neurons can express markers of multiple subtypes, we also considered two cholinergic markers [choline acetyltransferase (ChAt) and acetylcholine esterase (AChE)] and two glutamatergic markers [vesicular glutamate transporter (vGlut1) and glutamate decarboxylase (GadD1)]. First, gene expression was analyzed using semi-quantitative methods. Specifically, after RNA extraction, reverse transcription and PCR, the amplified cDNA was analyzed by gel electrophoresis. As shown in Fig 5, undifferentiated wild-type SH-SY5Y cells expressed all of the DA/NAergic markers. Nevertheless, the intensity of the bands representing *TH* and *VMAT2* expression were much weaker in comparison to the other markers, while the *DβH*-corresponding band was the most intense. Markers of both cholinergic and glutamatergic phenotypes were also expressed in undifferentiated SH-SY5Y cells. Cells differentiated with both TPA and RA did not exhibit clear differences in DAergic gene expression compared to untreated cells, while both cholinergic and glutamatergic markers appeared slightly upregulated. In contrast, upon staurosporine treatment the only appreciable difference was the upregulation of *VMAT2* expression. The same analysis performed on BE(2)-M17 cells revealed interesting differences (Fig 5). First, the expression of cholinergic and glutamatergic markers was extremely low in comparison to DAergic markers in both the undifferentiated and differentiated BE(2)-M17 cells. Second, cholinergic and glutamatergic markers had very low expression levels compared with their expression levels in SH-SY5Y cells. The intensity of the bands corresponding to *TH* and *VMAT2* were much more intense compared to the SH-SY5Y cells. Finally, the expression of *VMAT2* appeared to be upregulated following staurosporine treatment. Overall, these data indicate a more pronounced CAergic phenotype for BE(2)-M17 cells compared with SH-SY5Y cells. To obtain precise quantification of the effects of differentiation on the CAergic pathway in both SH-SY5Y and BE(2)-M17 cells, we performed qRT-PCR experiments.

**Fig 6:**
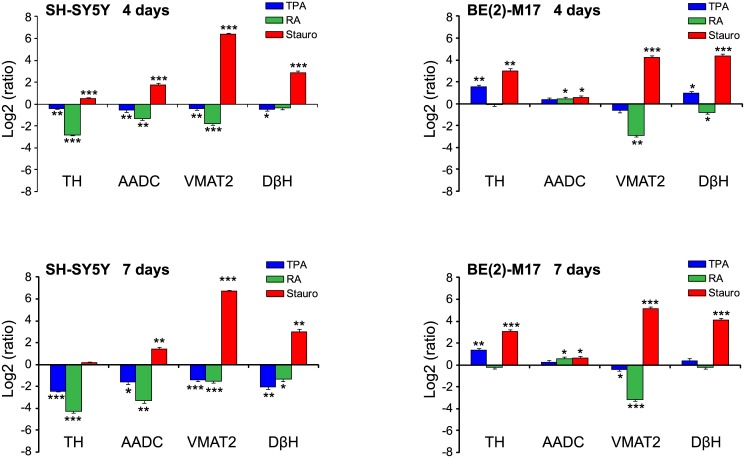
Gene expression profile of CAergic markers in differentiated SH-SY5Y and BE(2)-M17 cells. After 4 and 7 days of differentiation with TPA, RA and staurosporine, TH, AADC, VMAT2 and DβH mRNA levels were compared with their corresponding levels in undifferentiated cells using qRT-PCR. Expression was displayed on a Log_2_ scale. Positive and negative values indicated up and downregulation of the genes with respect to control cells (undifferentiated cells), respectively. For each gene, differences between differentiated and undifferentiated cells were tested for significance using Student’s t-test. (*P<0.*05*, **P<0.*01*, ***P<0.*001)*.

The results obtained after the addition of the differentiating agents are summarized in Table 2 and plotted in Fig 6. In SH-SY5Y cells, the expression profiles of CA-related genes 4 days after TPA treatment were only slightly different from the undifferentiated controls, with all genes slightly down-regulated relative to the control. This trend was more evident after 7 days of TPA treatment. We observed a trend of general down-regulation when the cells were treated with RA, with the effects visible as early as 4 days after treatment. Analysis at 7 days confirmed that the *TH* and *AADC* genes were the most affected, suggesting that DA synthesis was specifically inhibited. In contrast, cells treated with staurosporine exhibited an upregulation of CA-related markers at both 4 and 7 days after differentiation. Interestingly, while the expression of the *TH* gene was only slightly increased, *VMAT2* expression was increased by more than 80- and 100-fold at 4 and 7 days, respectively. Expression of the *DβH* gene was also considerably enhanced (~7- and ~8–fold at 4 and 7 days, respectively). Overall, the results indicated that while the machinery for DA synthesis appeared to be only slightly increased by staurosporine-induced differentiation, DA storage inside vesicles and its subsequent conversion into NA was clearly favored.

**Table 2:** Gene expression profile of the primary catecholaminergic markers after differentiation.

	SH-SY5Y				BE(2)-M17			
**TPA**	*TH*	*AADC*	*VMAT2*	*DβH*	*TH*	*AADC*	*VMAT2*	*DβH*
**4 days**	0.75±0.02	0.68±0.08	0.74±0.04	0.73±0.05	3.0 ±0.2	1.30±0.10	0.67±0.08	1.93±0.19
**7 days**	0.18±0.06	0.33±0.04	0.38±0.03	0.25±0.03	2.6±0.2	1.19±0.10	0.75±0.06	1.31±0.15
**RA**	*TH*	*AADC*	*VMAT2*	*DβH*	*TH*	*AADC*	*VMAT2*	*DβH*
**4 days**	0.14±0.03	0.40±0.03	0.30±0.01	0.79±0.05	0.93±0.06	1.34±0.08	0.14±0.01	0.58±0.05
**7 days**	0.05±0.02	0.10±0.01	0.34±0.02	0.40±0.04	0.85±0.05	1.48±0.12	0.11±0.01	0.87±0.07
**Stauro**	*TH*	*AADC*	*VMAT2*	*DβH*	*TH*	*AADC*	*VMAT2*	*DβH*
**4 days**	1.42±0.03	3.3±0.2	83±3	7.3±0.4	7.9±1.5	1.49±0.09	19.1±1.5	21±2
**7 days**	1.12±0.03	2.7±0.3	105±2	7.9±1.0	8.2±0.6	1.54±0.11	36±2	16.8±1.1

The effects observed in the BE(2)-M17 cell line after differentiation were less straightforward. Generally, the expression profiles observed after 4 and 7 days of treatment were very similar for each differentiating agent used. In the presence of TPA, the effects observed were rather mild (with the exception of the *TH* gene, which was upregulated ~3-fold) and the CAergic phenotype did not seem to change compared to undifferentiated cells. Even in the presence of RA the expression profiles of CA-related genes were only slightly different from the undifferentiated control, with the exception of the VMAT2 gene that was down-regulated 6-10-fold. As reported above for the SH-SY5Y cell line, staurosporine was the only agent among the differentiating agents tested that increased the CAergic phenotype of the BE(2)-M17 cells. Nevertheless, the *TH* gene was also strongly upregulated (~8-fold) in addition to the *VMAT2* and *DβH* genes.

In conclusion, our analysis of the effects induced on the CAergic pathway by cell differentiation indicated an enhancement of the NAergic phenotype both in SH-SY5Y and BE(2)-M17 cells after treatment with staurosporine. TPA and RA induced a loss of the CAergic phenotype in SH-SY5Y cells, although this effect was less evident in the BE(2)-M17 cells.

### Quantification of catecholamine levels

As reported in S2 File, we performed a Western blot analysis to assess the variations induced by differentiation in the expression level of the proteins involved in CA synthesis and storage. Nevertheless, because of the scanty reliability of some of the available antibodies, the results were not straightforward. As the quantification of the intracellular levels of DA and NA could represent a direct evaluation of the protein activity in defining the CAergic phenotype, we then decided to quantify the intracellular amounts of DA and NA in SH-SY5Y and BE(2)-M17 cells before and after differentiation, using HPLC coupled with an electrochemical detector. The DA and NA intracellular contents detected in the undifferentiated SH-SY5Y cells were 0.7 ± 0.1 and 1.7 ± 0.6 nmol (per gram of proteins), respectively. The DA levels were below the level of detection after a 7-day treatment with either TPA or RA, while the concentration of NA decreased to 0.4 ± 0.2 and 0.2 ± 0.1 nmol/g, respectively. This result is in agreement with our gene expression analysis and indicates a loss of the CAergic phenotype. Conversely, following the 7-day treatment with staurosporine the DA and NA levels were augmented to 2.4 ± 0.6 and 11 ± 3 nmol/g, respectively, indicating that differentiation promoted a more marked NAergic phenotype. The amounts of DA and NA detected in undifferentiated BE(2)-M17 cells were 9.2 ± 1.2 and 5.4 ± 1.7 nmol/g, respectively. Treatment with TPA for 7 days increased the levels of both DA and NA (11.3 ± 1.9 and 17.7 ± 3.0 nmol/g, respectively), which was in line with the modest upregulation of the *TH* and *DβH* genes observed through qRT-PCR. Moreover, in agreement with our gene expression analysis the CA content was not significantly modified by RA treatment, while the presence of staurosporine induced a strong increase in both the DA and NA concentrations. These results are summarized in Table 3.

**Table 3:** Cathecolamine contents detected in SH-SY5Y and BE(2)-M17 cell lines before and after differentiation for 7 days.

	DA	NA
**SH-SY5Y undifferentiated**	0.7±0.1	1.7±0.6
**SH-SY5Y with TPA**	nd	0.4±0.2
**SH-SY5Y with retinoic acid**	nd	0.2±0.1
**SH-SY5Y with staurosporine**	2.4±0.6	11±3
**BE(2)-M17 undifferentiated**	9.2±1.2	5.4±1.7
**BE(2)-M17 with TPA**	11.3±1.9	18±3
**BE(2)-M17 with retinoic acid**	9.7±1.5	7.5±1.5
**BE(2)-M17 with staurorine**	40±12	45±14

## Discussion

Cellular models are instrumental for *in vitro* or *ex vivo* studies to analyze the cellular pathways that govern physiological or pathological processes or to evaluate the cell toxicity or protection induced by different compounds, including potential drugs. While human induced pluripotent stem cells are emerging as the most suitable model, they are still mostly inaccessible for many research groups. As a consequence, it is germane to define robust criteria to choose the most suitable model from the several cellular models available to address the scientific question posed by the object of investigation. To provide one of these criteria, here we investigated the CAergic pathway of two human neuroblastoma cell lines: SH-SY5Y and BE(2)-M17. The first cell line has been largely studied and used in the field of neuroscience; therefore, it was chosen in this study for comparison; the second cell line has been poorly characterized to date and has emerged as a new experimental paradigm with a different CAergic phenotype.

Although undifferentiated SH-SY5Y and BE(2)-M17 cells are used in some circumstances, their differentiation towards a more neuronal phenotype is often preferred. For example, because undifferentiated cells are continuously dividing, their replication during the course of the experiment makes it difficult to unravel the extent to which neuroprotective or neurotoxic molecules influence the proliferation or cell degeneration rate, respectively [24]. For this reason, in the first part of this study we focused on the effects induced by three differentiating agents (RA, TPA and staurosporine) on growth inhibition, cell morphology and the expression of neuronal markers. The mechanism whereby RA regulates neurite outgrowth and growth inhibition includes the regulation of the transcription of neurotrophin receptors [25], the involvement of the Wnt signaling pathways [26] and the participation of type II protein kinase A [27]. The differentiating effects of TPA and staurosporine are primarily mediated by protein kinase C (PKC) isoforms. Nevertheless, while TPA is a PKC activator, staurosporine is a potent PKC inhibitor, suggesting distinct roles for different PKC isoforms during neuronal differentiation [28–31]. Our results indicated that all of the analyzed chemicals affected cell proliferation. Nevertheless, staurosporine was the most efficient treatment at inhibiting cell growth in the SH-SY5Y cell line, while treatment with RA was the most effective with the BE(2)-M17 cell line. Moreover, while TPA resulted in the formation of few and short processes in each cell line, both RA and staurosporine promoted a complex network of neuritic extensions. Finally, our immunofluorescence analysis demonstrated that SH-SY5Y and BE(2)-M17 cells expressed both β-III-tubulin and neurofilament, which are two markers of mature neurons, following RA- and staurosporine-induced differentiation.

Having confirmed the capability of each cell line to differentiate and adopt a neuron-like phenotype, we investigated and compared the CAergic pathways of these cells before and after differentiation using expression profile analysis of the major genes involved in CA metabolism (i.e., *TH*, *AADC*, *VMAT2* and *DβH*). Our results in SH-SY5Y cells indicated that both RA and TPA promoted the loss of the CAergic phenotype. In contrast, staurosporine treatment resulted in upregulation of all CA-related genes (particularly *VMAT2* and *DβH*). Conversely, the effects of TPA and RA on BE(2)-M17 cells were less evident, although staurosporine induced the upregulation of the genes involved in CA metabolism. The next step would have been a Western blot analysis to detect the translation products of the analyzed genes, but the scanty reliability of some of the available antibodies hindered trustworthy quantification [32, 33]. Therefore, we reasoned that quantification of the intracellular levels of DA and NA could represent a direct evaluation of the protein activity in defining the CAergic phenotype. Our data showed that both DA and NA accumulated in the undifferentiated SH-SY5Y cells, with the level of NA higher than DA. Consistent with the increased expression of the *DβH* gene, we observed a large increase in NA content relative to the control cells upon staurosporine treatment. In conclusion, the SH-SY5Y cells exhibit a more marked NAergic phenotype that was further enhanced following staurosporine-induced differentiation. The results obtained with the BE(2)-M17 cells were very interesting. First, the CA amounts detected in the undifferentiated cells were considerably higher than those observed for SH-SY5Y cells, indicating a more pronounced CAergic phenotype. Despite the presence of DβH, which should transform the newly synthesized DA into NA, DA was not only detectable but its concentration was even higher than the NA concentration. It is worth mentioning that high levels of DA were detected even though high DβH activity was measured also in the SK-N-BE(2) cells from which the BE(2)-M17 cells were subcloned [34]; this observation was consistent with our results. Furthermore, similar to the results obtained with the SH-SY5Y cells, we observed a large increase in both the DA and NA contents relative to the control cells following treatment with staurosporine.

In conclusion, our results emphasize that the two cell lines tested possess similar abilities to differentiate and acquire a neuron-like morphology. The most evident effects in the SH-SY5Y cells were observed in the presence of staurosporine, while in the BE(2)-M17 cells RA induced the strongest effects. Interestingly, relevant differences between the two cell lines were observed concerning the CAergic pathway. The basal expression of both DA and NA in undifferentiated cells was much more elevated in BE(2)-M17 cells, indicating that this cell line possesses a more pronounced CAergic phenotype compared with SH-SY5Y cells. Moreover, BE(2)-M17 cells have a more prominent DAergic phenotype, which makes the use of this cell line more suitable for studies focused on Parkinson’s disease, where the presence of DA appears to be an important requirement for a cell model. The use of these cells (both undifferentiated or after differentiation) should be avoided when the CAergic phenotype is not required (e.g., studies concerning Alzheimer’s disease). In this last example, the use of SY-SY5Y cells after differentiation using either RA or TPA appears to be much more appropriate.

## Supporting Information

S1 File Flow cytofluorimetric analysis. (PDF)

S2 File Western blot analysis. (PDF)
