# Supplementary material for: Analysis of the Catecholaminergic Phenotype in Human SH-SY5Y and BE(2)-M17 Neuroblastoma Cell Lines upon Differentiation
Source: PLoS One. 2015 Aug 28;10(8):e0136769. doi: 10.1371/journal.pone.0136769 (PMC4552590; doi:10.1371/journal.pone.0136769)
Supplement: S1 File — (PDF) [file pone.0136769.s001.pdf]

To detected cell death, Annexin V/PI double staining kit (eBiosciencesDx) has been used in flow cytofluorimetric analyses. The Annexin V corresponding signal provides a very sensitive method for detecting cellular apoptosis, while propidium iodide (PI) is used to detect necrotic or late apoptotic cells, characterized by the loss of the integrity of the plasma and nuclear membranes.

The data generated by flow cytometry are plotted in two-dimensional dot plots in which PI is represented versus Annexin V-FICT. These plots can be divided in four regions corresponding to: 1) viable cells which are negative to both probes (PI/FITC -/-; Q3); 2) apoptotic cells which are PI negative and Annexin positive (PI/FITC -/+; Q1); 3) late apoptotic cells which are PI and Annexin positive (PI/FITC +/+; Q2); 4) necrotic cells which are PI positive and Annexin negative (PI/FITC +/-; Q4).

As represented in Supp Fig.1, for both the SH-SY5Y and BE(2)-M17 cells, plots corresponding to wild-type cells are very similar to those recorded after cell differentiation while a slight difference is present between the two cell lines. For each condition tested 10,000 cells were analyzed and the percentage of cells in the Q1-Q4 regions are summarized in Supp Tab1. From the calculated values corresponding to viable, apoptotic and necrotic cells, it follows that none of the differentiating agents analyzed in this work at the experimental concentrations used induced apoptosis or necrosis.

Supp. Tab.1. Percentage of viable (Q3), apoptotic (Q1), late apoptotic (Q2) and necrotic (Q4) cells measured by flow citofluorimetry before and after differentiation.

	**SH-SY5Y**				**BE(2)-M17**			
	CNTR	*TPA*	*RA*	*Stauro*	*CNTR*	*TPA*	*RA*	*Stauro*
**Q1**	1%	2%	3%	2%	6%	5%	4%	3%
**Q2**	2%	5%	7%	4%	2%	4%	1%	1%
**Q3**	96%	91%	88%	91%	82%	83%	88%	91%
**Q4**	1%	2%	2%	3%	10%	8%	7%	5%

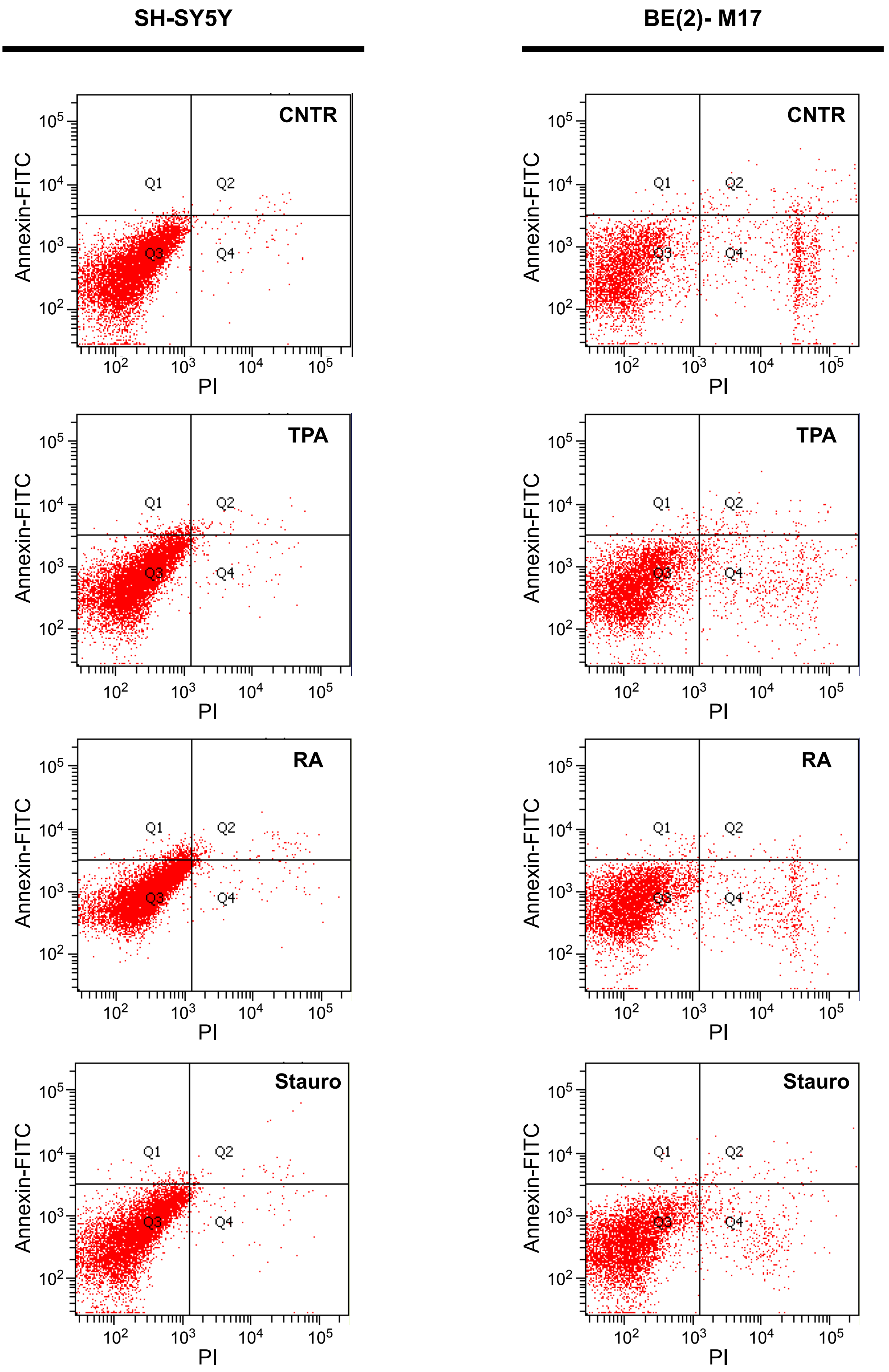

Supp. Fig. 1. Flow cytofluorimetric analysis performed on the SH-SY5Y and the BE(2)-M17 cells before and after differentiation.
