# Supplementary material for: Analysis of the Catecholaminergic Phenotype in Human SH-SY5Y and BE(2)-M17 Neuroblastoma Cell Lines upon Differentiation
Source: PLoS One. 2015 Aug 28;10(8):e0136769. doi: 10.1371/journal.pone.0136769 (PMC4552590; doi:10.1371/journal.pone.0136769)
Supplement: S2 File — (PDF) [file pone.0136769.s002.pdf]

To assess the variations induced by differentiation in the expression level of the proteins involved in CA synthesis and storage, we performed a Western blot analysis. The primary antibodies used included: goat anti-TH (1:900, Sigma SAB2501155), rabbit anti-AADC (1:900, Abcam ab131282), rabbit anti-VMAT2 (1:200, Sigma V9014) and rabbit anti-DβH (1:200, Santa Cruz H213). Mouse anti-GADH (1:10000, Origene TA150046) was used as reference. Among these proteins, we were unable to detect VMAT2, not even if the antibody was used at a very high concentration and the exposure time was extended overnight. Only nonspecific bands were visible on the developed film. Consistent with our results, in a recent work the specificity of many anti- and the VMAT2 antibodies was evaluated and the results indicated that most of them failed to detect the protein [1]. As in the previous work the antibody that we used in our analysis was not tested, we cannot exclude that the protein levels were below the detection limit. Concerning the other protein analyzed, as shown in Supp Fig.2, undifferentiated SH-SY5Y and BE(2)-M17 cells expressed all the CA markers with the exception of DβH, which was not visible in the BE(2)-M17 cell line. Upon differentiation, the expression levels of both TH and AADC were not significantly different from the wild-type cells. In contrast, upon staurosporine treatment the levels of DβH were strongly enhanced in the SH-SY5Y cells and a faint band also appeared in the BE(2)-M17cell line. This result is consistent with our gene-expression profile analysis.

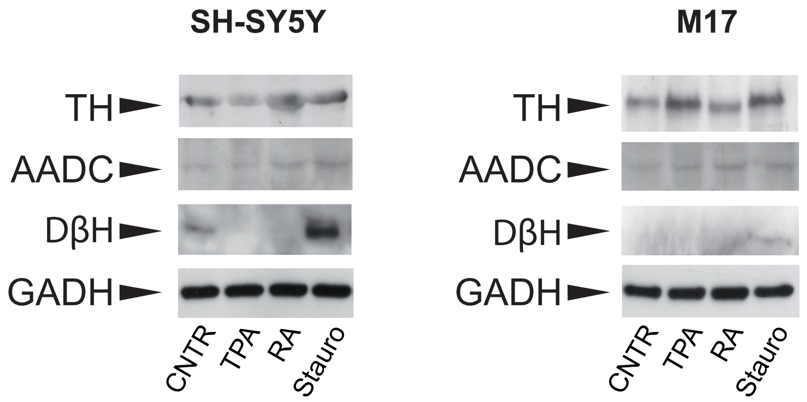

Supp. Fig. 2. Western blot analysis performed on the SH-SY5Y and the BE(2)-M17 cell lines before and after differentiation.

Supporting reference

1. Zhang S, Qi J, Li X, Wang HL, Britt JP, Hoffman AF, et al. Dopaminergic and glutamatergic microdomains in a subset of rodent mesoaccumbens axons. Nat Neurosci. 2015;18(3):386-92. Epub 2015/02/11. doi: 10.1038/nn.3945. PubMed PMID: 25664911; PubMed Central PMCID: PMC4340758.
